# Hydrogenations without Hydrogen: Titania Photocatalyzed Reductions of Maleimides and Aldehydes

**DOI:** 10.3390/molecules190915324

**Published:** 2014-09-24

**Authors:** David W. Manley, Luca Buzzetti, Andrew MacKessack-Leitch, John C. Walton

**Affiliations:** EaStCHEM School of Chemistry, University of St. Andrews, St. Andrews, Fife KY16 9ST, UK; E-Mails: dm53@st-andrews.ac.uk (D.W.M.); luca.buzzetti01@gmail.com (L.B.); acml2394@gmail.com (A.M.-L.)

**Keywords:** photocatalysis, reduction, titania, maleimides, aldehydes

## Abstract

A mild procedure for the reduction of electron-deficient alkenes and carbonyl compounds is described. UVA irradiations of substituted maleimides with dispersions of titania (Aeroxide P25) in methanol/acetonitrile (1:9) solvent under dry anoxic conditions led to hydrogenation and production of the corresponding succinimides. Aromatic and heteroaromatic aldehydes were reduced to primary alcohols in similar titania photocatalyzed reactions. A mechanism is proposed which involves two proton-coupled electron transfers to the substrates at the titania surface.

## 1. Introduction

Semiconductor photoredox catalysis (SCPC) with titania under aerobic conditions is a well-established method for homolytic oxidations and complete substrate mineralizations [1,2,3]. A few recent studies have shown that, under *anaerobic* conditions, TiO_2_ SCPC can also be adapted for constructive procedures that build organic structures. Examples include additions of enol-ethers to appropriate acceptors [4,5] and addition-cyclizations of aromatic amines with alkenes [6,7,8]. We recently discovered that TiO_2_ SCPC with carboxylic acid precursors could result in dimerizations and, with suitable alkene acceptors, both alkylations and tandem addition-cyclizations could be accomplished [9,10].

In our studies of TiO_2_ promoted reactions of a range of maleimides with carboxylic acids we observed that the alkylation products were always accompanied by significant amounts of the hydrogenated succinimides [10]. Absorption of a UV photon by the TiO_2_ particle leads to the creation of an electron/hole pair (e^−^/h^+^) which migrates to the surface. Hole capture by the carboxylic acid led to generation of the *C-*centered radical that alkylated the maleimide. It seemed probable, therefore, that the maleimide also played the role of an electron sink, consuming electrons in two sequential reduction-protonations, as well as acting as the radical acceptor. This gave us the idea that it might be possible to design a TiO_2_ based SCPC protocol for hydrogenating maleimides and also other electron-deficient unsaturated organic compounds. Such a procedure would offer several substantial advantages. The TiO_2_ is cheap and non-toxic and can easily be removed by filtration at the end of a reaction and re-cycled. Only soft UVA light (320–400 nm) is required, so standard Pyrex glassware can be used with ordinary sunlamp sources. Potentially, the process would be mild and environmentally friendly. In this paper, we describe the development of such a procedure and its successful application to hydrogenations of maleimides and carbonyl compounds.

## 2. Results and Discussion

### 2.1. SCPC Reductions of Maleimides

*N*-Phenylmaleimide (2) has strong electron-withdrawing character, is symmetrical, photostable and its photodimerization is negligible (under our experimental conditions). We employed this substrate during initial investigations to test the viability of the process. Previous SCPC experiments in our lab had shown that Aeroxide (Evonik) P25 TiO_2_, consisting of a 3:1 anatase/rutile mixture (particle size ~21 nm, surface area 50 m^2^·g^−1^, anatase excitation wavelength 385 nm, band gap 3.2 eV) was a good choice of catalyst. Water and oxygen led to oxidative degradations so they were excluded by using anhydrous solvents and purging the reaction mixtures with argon. For convenience Schlenk tubes were used as reactors and were stirred and irradiated with light from two semi-circular banks of Phillips sun-tanning tubes.

**Scheme 1:**
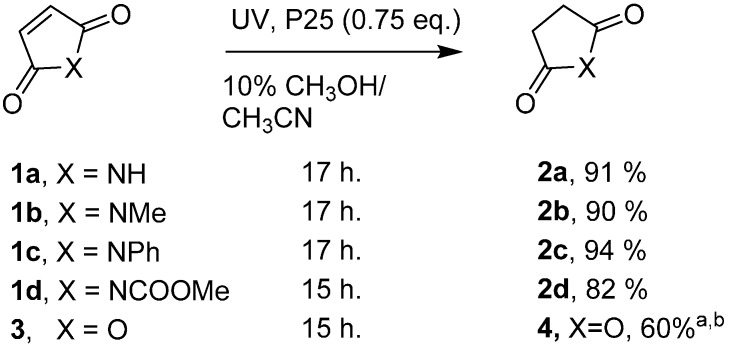
P25 promoted hydrogenations of commercial maleimides and maleic anhydride.

^a^ Yield determined by ^1^H-NMR; ^b^ 17% ring opened 4-oxobutanoic acid also observed.

When *N-*phenylmaleimide was irradiated alone in a dispersion of P25 in CH_3_CN poor conversion was observed. In the absence of a hole scavenger, the system was inefficient as the catalyst became electron deficient over time. When the reaction was repeated in methanol, a known hole-scavenger for TiO_2_, full conversion was achieved but nucleophilic ring-opening to the amide was observed to take place in competition with the reduction. Thus, as a compromise, a 10% mixture of CH_3_OH in CH_3_CN was trialed with P25 as a 1 mg·mL^−1^ dispersion (0.75 mole equiv.). This proved to be effective with **1c** being converted to *N-*phenylsuccinimide **2c** in a pleasing yield of 94%. This approach was extended to several commercially available maleimides and good to excellent yields (82%–94%) of the corresponding succinimides were obtained (Scheme 1). Only when the reduction was carried out using the highly electrophilic maleic anhydride **3** was a significant quantity of the ring opened product observed.

To extend the scope of the method, a set of maleimides with substituents in the amide and aromatic rings was prepared. An adaptation of the method due to Fles *et al.* [11] was used (Scheme 2). The substituted maleic anhydride (**3**) and a functionalized aromatic amine were refluxed in toluene for 3 days. The maleamic acid derivative prepared in this way was then cyclized by heating with acetic anhydride and sodium acetate to afford the required maleimides **5**, albeit in low yields.

**Scjeme 2:**
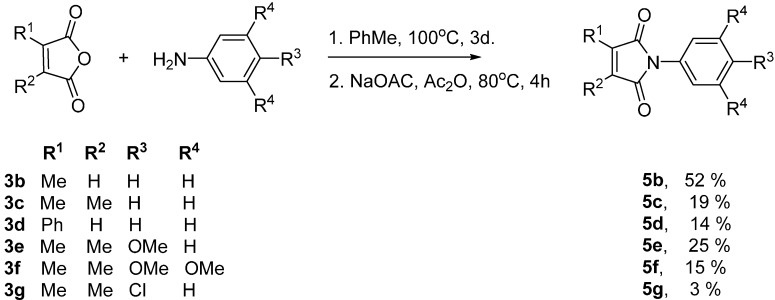
Preparation of functionalized maleimides.

The maleimides **5** were then subjected to SCPC reduction under the optimized conditions described above and all afforded the corresponding functionalized succinimides (pyrrolidine-2,5-diones) **6** (Table 1).

Lowest yields of the *N*-arylpyrrolidine-2,5-diones **6** were obtained for starting maleimides with single substituents in the maleimide rings. The 3,4-dimethyl precursor **5c** gave a better, but still very modest, yield. Satisfactory yields were obtained for the 3,4-dimethyl-substrates with the electron donating OMe substituents in the aromatic rings (**5e**,**5f**) but precursor **5g** with the electron-withdrawing 4*-*chloro-substituent also gave a poor product yield. Conversions were high in most cases and it is probable that significant product degradation occurred. Reduction of *N-*phenylphthalimide was also examined using the same conditions but, not surprisingly, no reduction of the aromatic ring took place.

**Table 1:** Titania semiconductor photoredox catalysis (SCPC) hydrogenations of substituted maleimides ^a^. 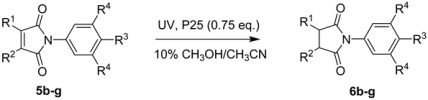

Substrate	R^1^, R^2^, R^3^, R^4^	Irrad. Time (h)	Yield of 6 (%)
**5b**	Me, H, H, H	20	≤5
**5c**	Me, Me, H, H	20	17
**5d**	Ph, H, H, H	20	9
**5e**	Me, Me, OMe, H	20	58
**5f**	Me, Me, OMe, OMe	72	79
**5g**	Me, Me, Cl, H	20	19

^a^ Yields determined by NMR.

### 2.2. SCPC Reductions of Carbonyl Compounds

**Scheme 3:**
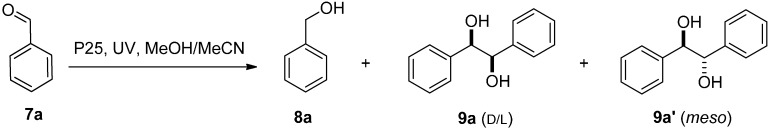
Products from the SCPC reaction of benzaldehyde.

Carbonyl compounds are also good electron acceptors and so SCPC reductions were investigated for a representative range. When dispersions of benzaldehyde and titania were irradiated in various solvents three products were observed, benzyl alcohol (**8a**) and the *meso* (**9a'**) and D/L (**9a**) diasterioisomeric set of 1,2-diphenylethane-1,2-diols (pinacols) (Scheme 3). Solvents containing 1%, 10%, 50% and 100% MeOH in MeCN were tested and the yield of **8a** was found to be a maximum for the 10% mixture.

**Figure 1:**
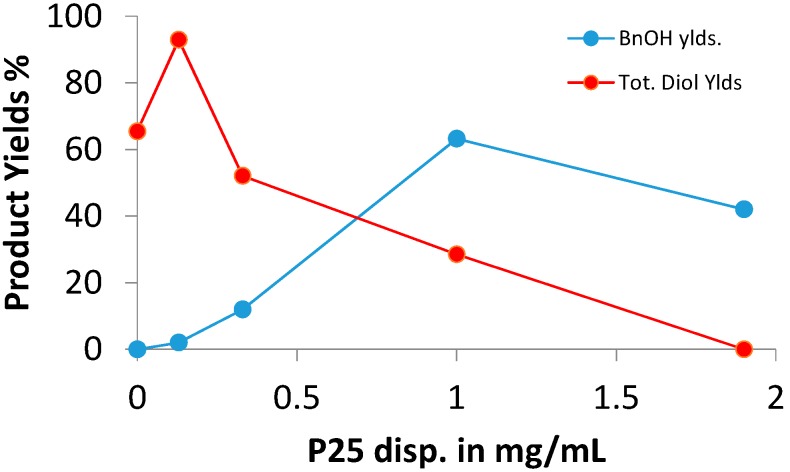
Product yields *vs.* P25 dispersion density in SCPC reactions of benzaldehyde.

The proportion of the alcohol to diol depended on the amount of P25 and the density of its dispersion within the reaction medium. A set of experiments with dispersions ranging from zero P25 to 1.9 mg·mL^−1^ was carried out and the yields of the two product types as a function of this are shown in Figure 1.

At light dispersions the process was selective for the diols which, as expected, were produced as a 1:1 mixture of the stereoisomers **9a** and **9a'**. The important result, however, was that irradiations with dense dispersions were more selective for reduction of the aldehyde to benzyl alcohol. To find the most efficient process a series of SCPC reactions was carried out with various catalysts. The same reaction conditions of solvent (MeCN and MeOH, 9:1), catalyst (0.75 mole equiv.) and irradiation time (20 h, except for zinc sulfide) were used for each system (Table 2).

**Table 2:** Product yields ^a^ from SCPC reactions of benzaldehyde with various catalysts.

Catalyst	BnOH (%)	Diols (9a + 9a') (%)	Ratio D/L: *meso*	Conversion (%)
P25	63	29	1.0	99
Pt-P25 ^b^	10	65	1.1	98
PC500	19	35	0.73	98
Coated Tube ^c^	22	49	1.0	99
ZnS ^d^	0	34	0.50	n.d.
ZnS ^e^	0	65	0.51	99

^a^ Yields determined by NMR; ^b^ P25 doped with Pt at loading of 0.1% (*w*/*w*); ^c^ NMR tube coated with anatase TiO_2_ by a sol gel process; ^d^ Irradiation time 1 h; ^e^ Irradiation time 3 h.

The platinized titania (Pt-P25) was prepared by coating P25 with 0.1% Pt using the method of Mills [12]. The Millenium PC500 (anatase, surface area ~300 m^2^·g^−1^, particle size 5–10 nm) had about six times the surface area of P25. The coated tubes were NMR tubes coated internally with a fine layer of anatase using the sol-gel process described by Mills and co-workers [13]. ZnS nanoparticles, prepared by using the method described by Hu, [14] have a more negative reduction potential than TiO_2_, (size 3–5 nm, *ca*. 156 m^2^·g^−1^ surface area) and so were also trialed. However, no reduction to benzyl alcohol took place with this catalyst. Table 2 shows that P25 was selective for hydrogenation and Pt-P25 was selective for dimerization. These catalysts also led to comparatively little product degradation and so were chosen for subsequent work.

It has been known since the earliest days of photochemistry that UV irradiation of carbonyl compounds produces pinacols [15]. The method was once widely exploited [16] but recent usage is scant [17]. Recent methods of production of 1,2-diols from carbonyl compounds rely on catalysis by metal complexes, notably of Ti [18,19,20,21]. The yields in Figure 1 show that 1,2-diol was formed by direct UVA photolysis, under our conditions, in the absence of catalyst. However, from the small increase in yield, it appeared at first that the presence of light dispersions of P25 or Pt-P25 might increase the efficiency of dimerization. Series of irradiations of a selection of aldehydes and ketones were carried out first with a light P25 dispersion (~0.25 mole equiv.) and then in the absence of catalyst with UVA alone (Table 3). To ensure that the NMR product identifications were correct, five of the reactions were scaled up and the product diols isolated and characterized (Table 3). This Table shows that no hydrogenation to alcohol took place with any of the carbonyl substrates in the absence of the P25 catalyst. The light dispersion of P25 gave very little alcohol and was selective for 1,2-diol formation for all the carbonyls that did react. Good to moderate yields of diols were obtained for all the substrates except the heterocycles and 2-naphthaldehyde. As expected, the diols were formed as a pair of stereoisomers (D/L and *meso*) in a ratio of 1:1 (within measurement error limits) in each case. Interestingly, the diol yields were greater in the absence of P25 for all the carbonyls except benzaldehyde. We conclude from this that diol formation took place by the well-known photochemical process and was independent of TiO_2_ catalysis.

**Table 3:** Comparison of carbonyl photolyses with and without P25 SCPC ^a^.

Carbonyl	Alcohol (%) with P25	Alcohol (%) UVA Only	Diol (%) with P25 ^b^	Diol (%) UVA Only
Benzaldehyde	0	0	93 {75}	65
2-Naphthaldehyde	0	0	0	0
4-Me-Benzaldehyde	7	0	43 {43}	72
4-MeO-Benzaldehyde	8	0	12	30
4-Cl-Benzaldehyde	5	0	54 {50}	58
4-CF_3_-Benzaldehyde	4	0	58 {49}	83
2-Thiophene-CHO	0	0	13	0
2-Furan-CHO	0	0	0	0
3-Benzofuran-CHO	8	0	0	0
Acetophenone	trace	-	66 {92}	-

^a^ Yields by NMR except as otherwise indicated; ^b^ Isolated yields in parenthesis {%}.

The benzaldehyde reaction (Figure 1) implied that carbonyls could be reduced to alcohols by use of denser dispersions of P25. Accordingly, a set of aldehydes and ketones was irradiated (20 h) in MeCN/MeOH (9:1) solvent with a P25 dispersion of 1 mg·mL^−1^ (~0.75 equiv.) and the product yields are recorded in Table 4.

**Table 4:** P25 SCPC reductions of carbonyl compounds to primary alcohols ^a^.

Carbonyl	Alcohol (%)	Diol (%)
benzaldehyde	63	29
2-naphthaldehyde	95	0
4-Me-benzaldehyde	36	16
4-MeO-benzaldehyde	56	0
4-Cl-benzaldehyde	78	21
4-CF_3_-benzaldehyde	76	0
2-thiophene-CHO	63	29
3-benzofuran-CHO	96	0
Acetophenone	0	6

^a^ Yields mol% by NMR.

Table 4 shows that TiO_2_ SCPC reductions of aldehydes to primary alcohols could be accomplished in good to excellent yields for aromatic and heterocyclic aldehydes with both electron-withdrawing and electron-releasing substituents. The selectivity for alcohol over 1,2-diol production was more variable. It is probable this selectivity could have been increased by using denser dispersions of P25. However, denser dispersions screen out the UVA and this leads to lower overall yields either because of incomplete substrate consumption, or because of product degradation if longer irradiation times were employed. The SCPC reductions of ketones to secondary alcohols were not successful. With acetophenone (Table 4) and benzophenone very little reaction took place and essentially only minor amounts of diols were observed; cyclohexanone also gave no cyclohexanol product.

In the absence of TiO_2_, absorption of a photon by the aldehyde generates a singlet diradical that crosses over to the triplet (Figure 5). The triplet abstracts an H-atom from solvent (SH) to afford hydroxy-benzyl type radical **10**. Two of these then combine to produce the 1,2-diol **9** as a 1:1 mixture of stereo-isomers. The lower portion of Figure 5 shows a schematic of a plausible surface process in the presence of TiO_2_ catalyst. Absorption of a photon by the TiO_2_ generates an e^−^/h^+^ pair that migrates to the surface of the catalyst particle. The TiO_2_ surface is plentifully populated with OH groups [22,23] which, if deuteriated CD_3_OD is used, will rapidly exchange to OD groups. The aromatic aldehyde H-bonds to the surface (**11**) where it readily accepts e^−^ from the e^−^/h^+^ pair (**12**). The methanol from the solvent also H-bonds to the surface (shown deuteriated in **11**) where it donates an electron to a surface h^+^ and also exchanges H for D as the radical cation is generated (**13**).

**Scheme 4:**
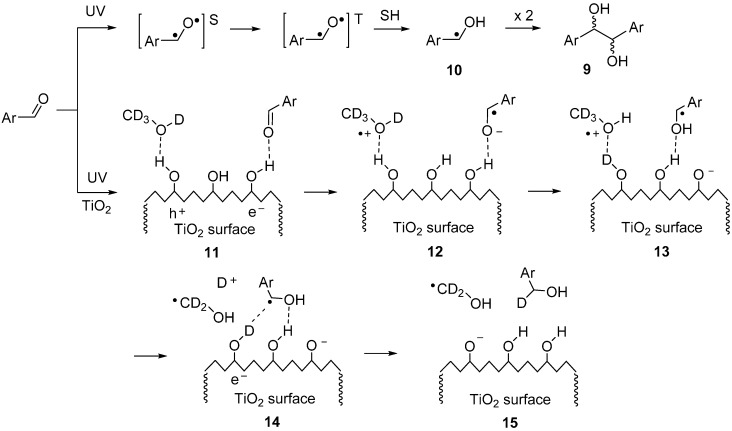
Proposed surface mechanism for the TiO_2_ SCPC reduction of aryl aldehydes.

A surface proton (or deuteron) is transferred to the aryl radical anion in **12** thus generating the resonance stabilized hydroxybenzyl radical in **13**. The latter accepts a second e^−^ from the TiO_2_ and the resulting benzyl anion picks up a deuteron (or proton) from a neighboring surface OD(H) (**14**). Finally, the benzyl type alcohol is released probably along with an hydroxymethyl radical.

We carried out an SCPC reduction of benzaldehyde using P25 and the conditions of Table 4 except that CD_3_OD replaced the CH_3_OH in the solvent mixture. The ^1^H-NMR spectrum of the product benzyl alcohol showed δ_H_ at 4.66 ppm (1:1:1 triplet, partly resolved *J ≈* 2 Hz) and the ^2^H-NMR spectrum showed δ_D_ at 4.66 ppm (d, *J* ≈ 2 Hz) consistent with the structure PhCHDOD. The GC-MS supported this structure in that the EI MS of the benzyl alcohol peak showed *m/z* 110 (M^+^). This result was good evidence in support of the mechanism shown in Scheme 4.

## 3. Experimental Section

### 3.1. General Procedures

All solvents and reagents were purchased from commercial sources and used without further purification unless stated otherwise. The acetonitrile and methanol were both distilled over calcium hydride in an atmosphere of argon. Column chromatography was run using Silica 60A (particle size 40–63 µm) as the stationary phase. Thin layer chromatography was performed on pre-coated silica gel plates (0.20 mm thick, Sil G UV253, Macherey-Nagel, Düren, Germany) and observed under UV light. ^1^H- and ^13^C-NMR spectra were recorded using the Bruker AV 300 and Bruker AV II 400 instruments (Bruker, Karlsruhe, Germany). Chemical shifts are reported in parts per million (ppm) from low to high frequency and referenced to residual solvent peaks unless otherwise stated. NMR yields were calculated using a known volume of dibromomethane (2 µL) as an internal standard. Coupling constants (*J*) are reported in hertz (Hz). Standard abbreviations indicating multiplicity were used as follows: s = singlet, d = doublet, t = triplet, dd = double doublet, q = quartet, m = multiplet, b = broad. Mass spectrometry was carried out by the services at the EPSRC National Mass Spectrometry Service in Swansea, UK.

*Zinc Sulfide* was prepared in accordance with the procedure of Hu *et al.* [14]. Zinc acetate, thiourea and polyvinylpyrrolidone were refluxed for 3 h at 150 °C in ethylene glycol. The resulting suspension was then centrifuged to remove the nanoparticles, which were then dried overnight in a vacuum oven. The polyvinylpyrrolidone is used to give smaller particle sizes for the ZnS.

### 3.2. General Procedure for Preparation of Maleimides

The aromatic amine (2.5 equiv.) and substituted maleic anhydride (1 equiv.) were refluxed in toluene at 110 °C for 3 days. The mixture was then concentrated under reduced pressure and the intermediate was washed with hydrochloric acid (1 M), before being dissolved in acetic anhydride (35 mL) with sodium acetate (20 equiv.) and heated to 80 °C for 4 h. After cooling to room temperature the acetic anhydride as removed *in vacuo* and the remaining solid was partitioned between ethyl acetate (75 mL) and deionized water (3 × 100 mL). The combined organic extracts were then washed with saturated aqueous sodium bicarbonate (100 mL) and dried over magnesium sulfate. The solvent was removed under reduced pressure and the crude product was recrystallized from cyclohexane to give the desired product.

*3-Methyl-1-phenyl-1H-pyrrole-2,5-dione* (**5b**). Aniline (2.45 mL, 26.76 mmol) and methyl maleic anhydride (1.5 g, 13.38 mmol) in toluene (30 mL) were treated according to the general procedure. In the second step sodium acetate (6.51 g, 79.4 mmol) and the intermediate were dissolved in acetic anhydride (35 mL). After recrystallization in cyclohexane **5b** was obtained as an off-white solid (1.3 g, 52%). ^1^H-NMR (400 MHz, CDCl_3_): δ 2.14 (3H, d, *J* = 2.14 Hz), 6.52 (1H, s), 7.35–7.49 (5H, m).

*3,4-Dimethyl-1-phenyl-1H-pyrrole-2,5-dione* (**5c**). From aniline (0.92 mL, 9.93 mmmol) and dimethyl maleic anhydride (0.5 g, 3.97 mmol). After recrystallization in cyclohexane **5c** was obtained as an off-white solid (0.15 g, 19%). ^1^H-NMR (400 MHz, CDCl_3_): δ 2.09 (6H, s), 7.35–7.39 (3H, m), 7.45–7.50 (2H, m). Spectral data was consistent with literature [24].

*1,3-Diphenyl-1H-pyrrole-2,5-dione* (**5d**). From aniline (1.05 mL, 11.48 mmol) and *N*-phenyl maleic anhydride (1.0 g, 5.74 mmol). Following column chromatography on silica gel (eluent: 20%–40% EtOAc in petroleum 40/60) **5d** was obtained as an off-white solid (0.2 g, 14%). ^1^H-NMR (400 MHz, CDCl_3_): δ 6.91 (1H, s), 7.45–7.58 (8H, m), 8.00 (2H, m). Spectral data was consistent with literature [25].

*1-(4-Methoxyphenyl)-3,4-Dimethyl-1H-pyrrole-2,5-dione* (**5e**). From 4-methoxyaniline (1.22 g, 9.93 mmol) and dimethyl maleic anhydride (0.5 g, 3.97 mmol). After recrystallization from cyclohexane **4** was obtained as a pale orange solid (0.23 g, 25%). ^1^H-NMR (400 MHz, CDCl_3_): δ 2.0 (6H, s), 3.75 (3H, s), 6.90 (2H, d, *J* = 9.0 Hz), 7.17 (2H, d, *J* = 9.0 Hz). Spectral data was consistent with literature [26].

*3,4-Dimethyl-1-(3,4,5-trimethoxyphenyl)-1H-pyrrole-2,5-dione* (**5f**). From 3,4,5-trimethoxyaniline (1.09 g, 5.95 mmol) and dimethyl maleic anhydride (0.5 g, 3.97 mmol). Following column chromatography on silica gel (eluent: 20%–40% EtOAc in petroleum 40/60) **5f** was obtained as an off-white solid (0.17 g, 15%); mp. 141 °C. ^1^H-NMR (400 MHz, CDCl_3_): δ 2.09 (6H, s), 3.94 (6H, s), 3.96 (3H, s), 6.59 (2H, s). ^13^C-NMR (100MHz, CDCl_3_): δ 8.93, 56.17, 60.89, 77.35, 103.65, 127.51, 137.46, 153.35, 171.06. LR-ESIMS: *m/z* = 292 [M*H*]^+^; HR-EIMS: *m/z* = 292.1173 (calcd. for C_15_H_18_N_1_O_5_, 292.1179).

*1-(4-Chlorophenyl)-3,4-dimethyl-1H-pyrrole-2,5-dione* (**5g**). From 4-chloroaniline (2.0 g, 15.7 mmol) and dimethyl maleic anhydride (0.5 g, 3.97 mmol). Following column chromatography on silica gel (eluent: 20%–40% EtOAc in petroleum 40/60) **5g** was obtained as a pale brown solid (0.03 g, 3%); mp. 142 °C. ^1^H-NMR (400 MHz, CDCl_3_): δ 2.10 (6H, s), 7.24–7.26 (2H, m), 7.33–7.36 (2H, m). ^13^C-NMR (100 MHz, CDCl_3_): δ 8.94, 126.78, 129.19, 130.51, 133.21, 137.64, 170.62. LR-ESIMS: *m/z* = 236 [M*H*]^+^; HR-EIMS: *m/z* = 236.0472 (calcd. for C_12_H_11_Cl_1_N_1_O_2_, 236.0473).

### 3.3. General Procedure for SCPC Hydrogenation of Maleimides

The maleimide was added to a suspension of P25 in methanol (10%) and acetonitrile (90%). The resulting mixture was then purged with argon for 15 min. The mixture was irradiated with eight 29 cm 15 W Philips Cleo tubes (λ = 350 nm). Following irradiation the P25 powder was removed via filtration through Celite.

*Succinimide* (**2a**). Maleimide (24.5 mg, 0.25 mmol) and TiO_2_ (30 mg, 0.38 mmol) in CH_3_OH (3 mL) and CH_3_CN (27 mL) were reacted in accordance with the general procedure. Following irradiation for 17 h **2a** was obtained as a white powder (22.8 mg, 91%). ^1^H-NMR (300 MHz, CDCl_3_, 296 K): δ = 2.75 (s, 4H), 8.84 (br-s, 1H, N*H*); ^13^C-NMR (75 MHz, CDCl_3_, 299 K): δ = 29.6, 177.9. Consistent with literature [27].

*N-Methylsuccinimide* (**2b**). *N*-Methylmaleimide (27.8 mg, 0.25 mmol) and TiO_2_ (30 mg, 0.38 mmol) in CH_3_OH (3 mL) and CH_3_CN (27 mL) were reacted in accordance with the general procedure. Following irradiation for 17 h **2b** was obtained as an off white powder (25.5 mg, 90%). ^1^H-NMR (300 MHz, CDCl_3_, 298 K): δ = 2.69 (s, 4H), 2.97 (s, 3H); ^13^C-NMR (75 MHz, CDCl_3_, 299 K): δ = 24.8, 28.2, 177.3. Consistent with the literature [28].

*1-Phenylpyrrolidine-2,5-dione* (**2c**). *N*-Phenyl maleimide (0.0347 g, 0.2 mmol) and TiO_2_ (0.012 g, 0.15 mmol) were added to methanol (1.1 mL) and acetonitrile (10.9 mL). Following irradiation for 20 h, **2c** was obtained as an off white solid (0.33 g, 94%). ^1^H-NMR (400 MHz, CDCl_3_): δ 2.90 (4H, s), 7.20–7.22 (2H, m), 7.30–7.35 (1H, m), 7.37–7.43 (2H, m). ^13^C-NMR (75 MHz, CDCl_3_, 297 K): δ = 28.4, 126.5, 128.7, 129.2, 132.0, 176.3. Spectral data was consistent with literature [29].

*N-Carboxymethylsuccinimide* (**2d**). *N*-Carboxymethylmaleimide (38.8 mg, 0.25 mmol) and TiO_2_ (30 mg, 0.38 mmol) in CH_3_OH (3 mL) and CH_3_CN (27 mL) were reacted in accordance with the general procedure. Following irradiation for 15 h **2d** was obtained as colorless oil (32.1 mg, 82%). ^1^H-NMR (400 MHz, CDCl_3_, 296 K): δ = 2.81 (s, 4H), 3.96 (s, 3H); ^13^C-NMR (75 MHz, CDCl_3_, 299 K): δ = 28.6, 172.6, 176.4.

*Succinic anhydride* (**4**). Maleic anhydride (24.5 mg, 0.25 mmol) and TiO_2_ (30 mg, 0.38 mmol) in CH_3_OH (3 mL) and CH_3_CN (27 mL) were reacted in accordance with the general procedure. Following irradiation for 15 h, **4** was observed by ^1^H-NMR (60% w.r.t. a CH_2_Br_2_ standard). ^1^H-NMR (300 MHz, CDCl_3_, 296 K): δ = 3.00 (s, 4H). 4-Oxobutanoic acid was observed in the same manner (17%). ^1^H-NMR (300 MHz, CDCl_3_, 296 K): δ = 2.59–2.70 (m, 4H), 3.69 (s, 3H), 9.86 (br-s, 1H). Spectral data for both compounds consistent with literature [30].

*3-Methyl-1-phenylpyrrolidine-2,5-dione* (**6b**). From 3-methyl-1-phenyl-1H-pyrrole-2,5-dione (0.037 g, 0.2 mmol) and TiO_2_ (0.12 g, 0.15 mmol). Following irradiation for 20 h, **6b** was obtained as an off white solid, ^1^H-NMR (wrt CH_2_Br_2_ internal standard) revealed 97% conversion of the starting material and ≤5% yield of the product. ^1^H-NMR (400 MHz, CDCl_3_): δ 1.47 (3H, m), 2.55 (1H, m), 3.07–3.09 (1H, m), 3.15–3.19 (1H, m), 7.25–7.51 (5H, m). Spectral data was consistent with literature [31].

*3,4-Dimethyl-1-phenylpyrrolidine-2,5-dione* (**6c**). 3,4-Dimethyl-*N*-phenylmaleimide **5c** (0.0402 g, 0.2 mmol) and TiO_2_ (0.12 g, 0.15 mmol) were added to a solution of methanol (1.1 mL) and acetonitrile (10.9 mL). Following irradiation for 20 h, **6c** was obtained as an off white solid, ^1^H-NMR (wrt CH_2_Br_2_ internal standard) revealed 50% conversion of the starting material and 17% yield of the product. ^1^H-NMR (400 MHz, CDCl_3_): δ 1.45 (6H, d, *J* = 7.0 Hz), 2.60 (2H, m), 7.31–7.32 (2H, m), 7.35–7.37 (2H, m), 7.47–7.49 (2H, m). Spectral data was consistent with literature [26].

*1,3-Diphenylpyrrolidine-2,5-dione* (**6d**). From 1,3-diphenyl-1*H*-pyrrole-2,5-dione (0.049 g, 0.2 mmol) and TiO_2_ (0.12 g, 0.15 mmol). Following irradiation for 20 h, **6d** was obtained as an off white solid, ^1^H-NMR (wrt CH_2_Br_2_ internal standard) revealed 99% conversion of the starting material and 9% yield of the product. ^1^H-NMR (400 MHz, CDCl_3_): δ 3.02 (1H, dd, *J* = 23.4 and 4.8), 3.36 (1H, dd, *J* = 9.9 and 18.7 Hz), 4.2 (1H, m), 7.37–7.51 (10H, m).

*1-(4-Methoxyphenyl)-3,4-Dimethylpyrrolidine-2,5-dione* (**6e**). From 3, 4-dimethyl-*N*-4-methoxyphenyl maleimide (0.046 g, 0.2 mmol) and TiO_2_ (0.12 g, 0.15 mmol). Following irradiation for 20 h, **5e** was obtained as an off white solid, ^1^H-NMR (wrt CH_2_Br_2_ internal standard) revealed 64% conversion of the starting material and a 58% yield. ^1^H-NMR (400 MHz, CDCl_3_): δ 1.44 (6H, d, *J* = 6.9 Hz), 2.58 (2H, m), 3.84 (3H, s), 6.98 (2H, s), 7.19 (2H, s). Spectral data was consistent with literature. [26].

*3,4-Dimethyl-1-(3,4,5-trimethoxyphenyl)pyrrolidine-2,5-dione* (**6f**). From 3,4-dimethyl-*N*-(3,4,5-trimethoxyphenyl) maleimide (0.058 g, 0.2 mmol) and TiO_2_ (0.12 g, 0.15 mmol). Following irradiation for 72 h, **6e** was obtained as an off white solid, (0.048 g, 79%). ^1^H-NMR (400 MHz, (CD_3_)_2_CO): δ 0.92 (6H, d, *J* = 7.4 Hz), 2.82 (2H, dq, *J* = 2.9 and 16.8 Hz), 3.46 (6H, s), 3.48 (3H, s), 6.82 (2H, s).

*1-(4-Chlorophenyl)-3,4-dimethylpyrrolidine-2,5-dione* (**6g**). From 1-(4-chlorophenyl)-3,4-dimethyl-1H-pyrrole-2,5-dione (0.0235 g, 0.1 mmol) and TiO_2_ (0.06 g, 0.075 mmol). Following irradiation for 20 h **6g** was obtained as an off white solid, ^1^H-NMR analysis (wrt CH_2_Br_2_ internal standard) revealed 49% conversion of the starting material and a 19% yield; ^1^H-NMR (400 MHz, CDCl_3_): δ 1.34 (6H, d, *J* = 7.3 Hz), 3.13 (2H, dq, *J* = 2, 16 Hz), 7.33–7.35 (2H, m), 7.42–7.44 (2H, m).

### 3.4. General Procedure for SCPC Reduction of Carbonyl Compounds

An oven dried Pyrex Schlenk tube was evacuated while still hot and then back-filled with argon. This was repeated three times before the tube was allowed to cool to room temperature. The carbonyl compound was added to a suspension of P25 in methanol (10%) and acetonitrile (90%) with a fast stream of argon flowing before freshly distilled CH_3_CN was added to generate the desired dispersion density (typically 1 mg·mL^−1^ unless otherwise stated). The resulting mixture was purged with argon for 20 min. The stirred mixture was then irradiated while still under an atmosphere of argon with two hemispherical banks of six 29 cm 15 W Philips Cleo tubes (λ = 350 nm) for the desired reaction time at ambient temperature. Following irradiation the TiO_2_ powder was removed by filtration through a Celite pad and the solvent removed under reduced pressure. Isolated yields were obtained from scale-up reactions and, where indicated, yields were calculated from the ^1^H-NMNR spectra w.r.t. CH_2_Br_2_ as internal standard. Characterization data for the compounds prepared using this procedure follows.

*From Benzaldehyde.* Benzyl alcohol: ^1^H-NMR (500 MHz, CDCl_3_): δ 4.70 (2H, s), 7.24–7.38 (5H, m). 1.2-diphenylethane-1.2-diol (D/L): ^1^H-NMR (500 MHz, CDCl_3_): δ 4.72 (2H, s), 7.12–7.24 (10H, m). 1.2-diphenylethane-1.2-diol (*meso*): ^1^H-NMR (500 MHz, CDCl_3_): δ 4.8 4(2H, s), 7.12–7.24 (10H, m). Chemical shifts were in accordance with the literature [32].

*From 2-Naphthaldehyde*. (Naphthalen-6-yl)methanol: ^1^H-NMR (500 MHz, CDCl_3_): δ 4.86 (2H, s), 7.45–7.50 (3H, m), 7.80–7.86 (4H, m). Chemical shifts were in accordance with the literature [33].

*From 4-Methylbenzaldehyde.* 4-Methylphenylmethanol: ^1^H-NMR (500 MHz, CDCl_3_): δ 2.16 (3H, s), 4.64 (2H, s), 7.02–7.33 (4H, m). 1,2-Di(4-methylphenyl)ethane-1,2-diol (D/L): ^1^H-NMR (500 MHz, CDCl_3_): δ 2.16 (3H, s) 4.66 (2H, s), 7.02–7.33 (8H, m). 1,2-Di(4-methylphenyl)ethane-1,2-diol (*meso*): ^1^H-NMR (500 MHz, CDCl_3_): δ 2.16 (3H, s) 4.74 (2H, s), 7.02–7.33 (8H, m). Chemical shifts were in accordance with the literature [32,34].

*From 4-Methoxybenzaldehyde.* 4-Methoxyphenylmethanol: ^1^H-NMR (500 MHz, CDCl_3_): δ 3.84 (6H, s), 4.62(2H, s,), 6.75–7.17 (4H, m). 1,2-Di(4-methoxyphenyl)ethane-1,2-diol (D/L): ^1^H-NMR (500 MHz, CDCl_3_): δ 3.84 (6H, s), 4.64 (2H, s), 6.75–7.17 (8H, m). 1,2-Di(4-methoxyphenyl)ethane-1,2-diol (*meso*): ^1^H-NMR (500 MHz, CDCl_3_): δ 3.84 (6H, s), 4.73 (2H, s), 6.75–7.17 (8H, m). Chemical shifts were in accordance with the literature [35].

*From 4-Chlororbenzaldehyde.* 4-Chlorophenylmethanol: ^1^H-NMR (500 MHz, CDCl_3_): δ 4.65 (2H, s), 7.00–7.31 (4H, m). 1,2-Di(4-chlorophenyl)ethane-1,2-diol (D/L): ^1^H-NMR (500 MHz, CDCl_3_): δ 4.59 (2H, s), 7.00–7.31 (8H, m). 1,2-Di(4-chlorophenyl)ethane-1,2-diol (*meso*): ^1^H-NMR (500 MHz, CDCl_3_): δ 4.82 (2H, s), 7.00–7.31 (8H, m). Chemical shifts were in accordance with the literature [32,36].

*From 4-(Trifluoromethyl)benzaldehyde.* 4-(Trifluoromethyl)phenylmethanol: ^1^H-NMR (500 MHz, CDCl_3_): δ 4.67 (2H, s), 7.21–7.60 (4H, m). 1,2-Di(4-trifluoromethyl)diphenylethane-1,2-diol (D/L): ^1^H-NMR (500 MHz, CDCl_3_): δ 4.72 (2H, s), 7.21–7.60 (8H, m). 1,2-Di(4-trifluoromethyl)diphenylethane ethane-1,2-diol (*meso*): ^1^H-NMR (500 MHz, CDCl_3_): δ 4.95 (2H, s), 7.21–7.60 (8H, m). Chemical shifts were in accordance with the literature [37,38].

*From 2-Thiophenecarboxaldehyde*. Thiophene-2-methanol: ^1^H-NMR (500 MHz, CDCl_3_): δ 4.83 (2H, s), 7.81–7.28 (3H, m). 1.2-di(thiophenyl)ethane-1.2-diol (D/L): ^1^H-NMR (500 MHz, CDCl_3_): δ 5.06 (2H, s), 6.81–7.28 (6H, m). 1.2-di(thiophenyl)ethane-1.2-diol (*meso*): ^1^H-NMR (500 MHz, CDCl_3_): δ 5.13 (2H, s), 6.81–7.28 (6H, m). Chemical shifts were in accordance with the literature [36,39].

*From 2-Benzofurancarboxaldehyde*. 1-(Benzofuran-2-yl)methanol: ^1^H-NMR (500 MHz, CDCl_3_): δ 4.78 (2H, s), 6.40 (1H, s) 7.10–7.40 (6H, m). Chemical shifts were in accordance with the literature [40].

*From Acetophenone*. 1-Phenylethanol: ^1^H-NMR (500 MHz CDCl_3_): δ 4.75 (1H, s), 7.21–7.60 (4H, m). 2,3-Diphenylbutane-2,3-diol (D/L): ^1^H-NMR (500 MHz CDCl_3_): 1.39 (6H, s), 7.06–7.11 (10H, m). 2,3-Diphenylbutane-2,3-diol (*meso*): ^1^H-NMR (500 MHz CDCl_3_): 1.46 (6H, s), 7.06–7.11 (10H, m). Chemical shifts were in accordance with the literature [35].

## 4. Conclusions

Our exploratory study demonstrated that both electron-deficient alkenes and aldehydes could be effectively hydrogenated by P25 SCPC. The reactions took place at room temperature and required only non-harmful UVA irradiation and dry solvents. These are mild processes because no toxic metals or corrosive reagents, dangerous gases or forcing conditions are needed and the TiO_2_ catalyst could be recovered and re-cycled. They represent an important step towards greener synthetic routes.
